# Is intrinsic lumbar spine shape associated with lumbar disc degeneration? An exploratory study

**DOI:** 10.1186/s12891-020-03346-7

**Published:** 2020-07-03

**Authors:** Janet A. Deane, Anastasia V. Pavlova, Adrian K. P. Lim, Jennifer S. Gregory, Richard M. Aspden, Alison H. McGregor

**Affiliations:** 1 Sackler MSK LAB, Sir Michael Uren Hub, Imperial College London, White City Campus, 86 Wood Lane, London, W12 0BZ UK; 2 Imaging Department, Charing Cross Hospital, Imperial College Healthcare NHS Trust, London, UK; 3 Aberdeen Centre for Arthritis and Musculoskeletal Health, School of Medicine, Medical Sciences and Nutrition, University of Aberdeen, Aberdeen, UK

**Keywords:** Low back pain, Lumbar disc degeneration, Quality of life, MRI, Statistical shape modelling

## Abstract

**Background:**

Lumbar disc degeneration (LDD) is a condition associated with recurrent low back pain (LBP). Knowledge regarding effective management is limited. As a step towards the identification of risk, prognostic or potentially modifiable factors in LDD patients, the aim of this study was to explore the hypothesis that intrinsic lumbar spine shape is associated with LDD and clinical outcomes in symptomatic adults.

**Methods:**

3 T MRI was used to acquire T2-weighted sagittal images (L1-S1) from 70 healthy controls and LDD patients (mean age 49 years, SD 11, range 31–71 years). Statistical Shape Modelling (SSM) was used to describe lumbar spine shape. SSM identified variations in lumbar shape as ‘modes’ of variation and quantified deviation from the mean. Intrinsic shape differences were determined between LDD groups using analysis of variance with post-hoc comparisons. The relationship between intrinsic shape and self-reported function, mental health and quality of life were also examined.

**Results:**

The first 7 modes of variation explained 91% of variance in lumbar shape. Higher LDD sum scores correlated with a larger lumbar lordosis (Mode 1 (55% variance), *P* = 0.02), even lumbar curve distribution (Mode 2 (12% variance), *P* = 0.05), larger anterior-posterior (A-P) vertebral diameter (Mode 3 (10% variance), *P* = 0.007) and smaller L4-S1 disc spaces (Mode 7 (2% variance), *P* ≤ 0.001). In the presence of recurrent LBP, LDD was associated with a larger A-P vertebral diameter (Mode 3) and a more even lumbar curvature with smaller L5/S1 disc spaces (Mode 4), which was significantly associated with patient quality of life (*P* = 0.002–0.04, r_p_ = 0.43–0.61)).

**Conclusions:**

This exploratory study provides new evidence that intrinsic shape phenotypes are associated with LDD and quality of life in patients. Longitudinal studies are required to establish the potential role of these risk or prognostic shape phenotypes.

## Background

Lumbar disc degeneration (LDD) is a condition associated with recurrent low back pain (LBP) [1], the lifetime prevalence of which may be as much as 80% [2]. LDD is commonly evaluated using the Pfirrmann [3] and modified Pfirrmann grading systems [4], which use T2-weighted sagittal MR images of the intervertebral discs to grade the lack of distinction between the nucleus and annulus and the reduction in intervertebral disc height and signal intensity associated with LDD [3] .

Current treatment approaches for recurrent LBP offer small to moderate effects in terms of a sustained improvement in quality of life and disability [5, 6]. As a step towards a potential role of lumbar shape and Statistical Shape Modelling (SSM) in the identification of risk, prognostic or potentially modifiable factors in LDD patients, it is pressing to identify the phenotypes associated with LDD through the examination of observable traits. Since the external spinal curvature is not always representative of the internal spinal geometry [7], it seems appropriate to examine the internal architecture of the lumbar spine or intrinsic lumbar shape.

SSM is a statistical image analysis technique that, through the reduction of variables using principal components analysis, is used to describe and quantify variations in joint morphology and intrinsic shape [7, 8]. Conventionally, geometric measurements of lumbar lordosis are performed but these do not account for the distribution of lumbar curvature or morphological variability in the degenerate spine [9]. However, SSM can describe this and has been shown to be a more reliable and precise method of characterising lumbar curvature (4% measurement error) when compared with conventional measurements, such as the Cobb angle (10% error) [10].

To our knowledge, SSM has not been used to examine intrinsic lumbar shape in LDD adults.

The aim of this exploratory study is to create a shape model of the lumbar spine using SSM and to test the hypothesis that intrinsic lumbar spine shape is associated with LDD and clinical outcomes in adults.

## Methods

Seventy participants were recruited through advertisement from primary and secondary care between September 2015 and May 2017. Each participant provided informed consent and met strict inclusion and exclusion criteria (Table 1).

**Table 1:** Inclusion and exclusion criteria

**Healthy Participants**	**Inclusion Criteria**	**Exclusion Criteria**
	• ≥ 30 years • No low back pain • No recurrent history of low back pain • No episodes of LBP lasting greater • than 3 months duration	• Spinal surgery • Malignancy • Spondylolisthesis • Peripheral neuropathy with loss of sensation • Systemic or spinal infection • Neurological disease or balance disorder • Disorders affecting pain perception • Significant cardiovascular or metabolic disease • Severe musculoskeletal deformity (scoliosis, osteoporosis, Paget’s disease, fracture) • Spinal surgery or major surgery within three months prior to testing • MRI contraindicated
**Patients**	• ≥ 30 years • Evidence of LDD without neural compression on MRI • Recurrent low back pain (central/ unilateral) of greater than 3 months duration • MRI as part of routine NHS care	

A 3 T Verio MRI scanner (Siemens Medical Systems, Erlangen, Germany) was used to acquire T2-weighted sagittal lumbar spine images (L1-L5/S1) from the asymptomatic volunteers and symptomatic participants as part of routine NHS care. The majority of scans were acquired at the same time of day in supine crook lying supported by a wedge (with shoulders, thorax and pelvis level) following a 10 min rest period [11].

T2-weighted sagittal images were viewed using the Picture Archiving and Communications System (PACs) (Synpase, Fujifilm Medical Systems, Tokyo, Japan). Pfirrmann and Modified Pfirrmann disc grades were determined by an experienced consultant radiologist, blinded to the demographics and clinical data of participants. LDD sum scores were calculated through the summation of Pfirrmann scores from each lumbar disc [12]. Four groups were identified based upon the presence or absence of pain and LDD (modified Pfirrmann grade ≥ 6). Pain was determined by the presence or absence of recurrent LBP for greater than 3 months. Self-reported clinical outcome tools including the Short Form 36, Version 2 (SF-36), the Oswestry Disability Index (ODI) and the Hospital Anxiety and Depression Scale (HADS) were used to assess quality of life, function and anxiety and depression, respectively.

### Statistical shape modelling (SSM)

SSM is an image processing method designed to characterise the shape of an object within a series of image [8]. It uses principal components analysis to identify different ‘modes’ of variation (principal components) in sagittal spinal shape and computes mode scores (output variables) for each image to quantify that variation by calculating deviation from the mean of all the images [10, 11, 13].

**Fig. 1:**
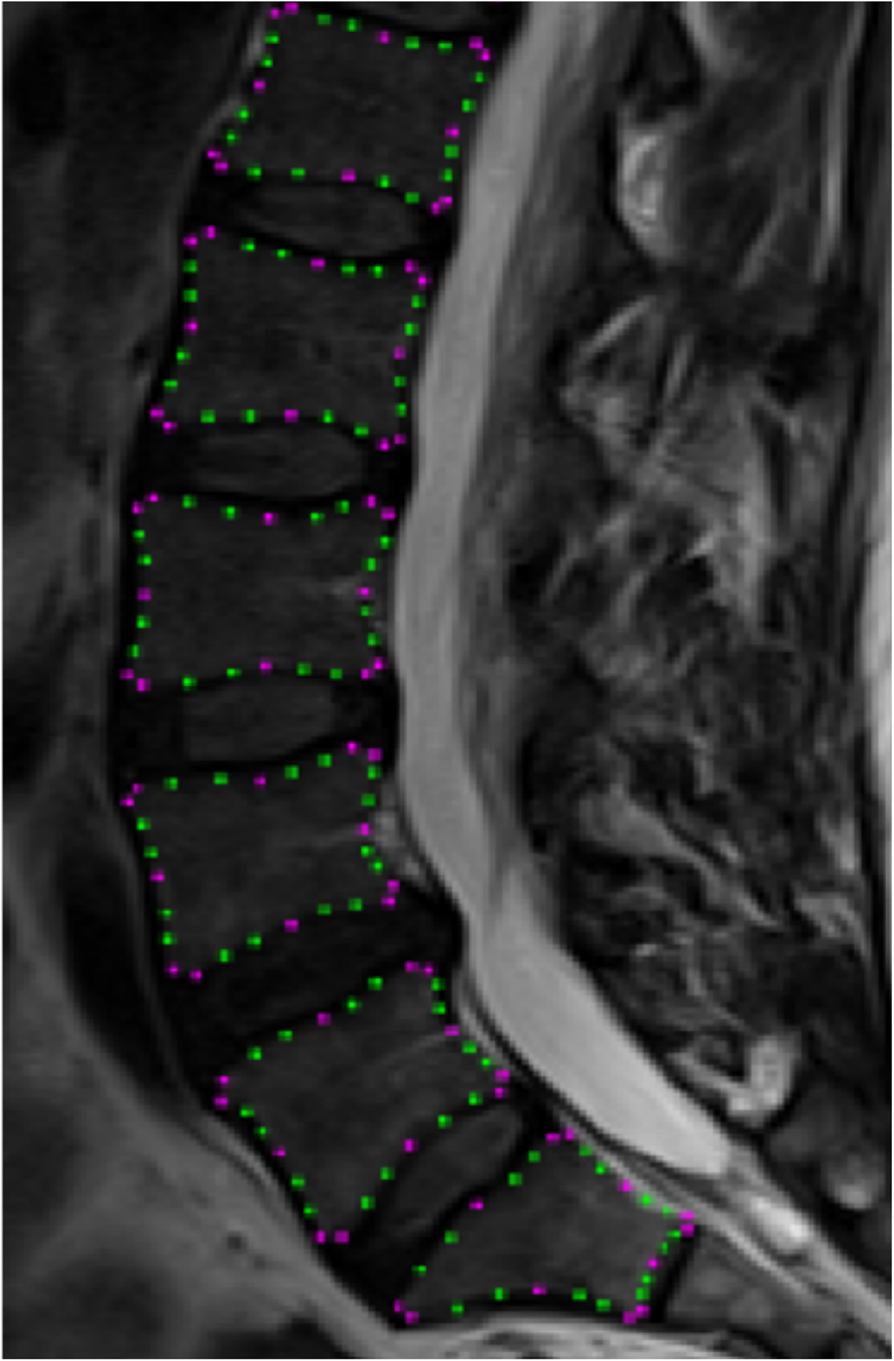
Statistical Shape Modelling (SSM) template of the lumbar spine. Sagittal T-2 weighted image the lumbar spine marked up using 168-point template (L1-S1). Key points are indicated in pink

Seventy consistent mid-sagittal slices (maximum spinal canal width) were selected from the participant’s data using Image J software and exported as Bitmap image files. A previously tested 168-point lumbar spine template [10, 14] which defined the outline of each vertebral body from the 1st lumbar to 1st sacral vertebrae (L1-S1) was recreated in custom SSM software (Shape software, University of Aberdeen) and images uploaded for analysis. In each image, key points were identified manually by the same tester and the point template fitted to the spine (Fig. 1). Intra-class correlation coefficients (ICC) demonstrated excellent reliability of key point positioning of both x (ICC = 0.99) and y (ICC = 0.98) co-ordinates. The influence of positioning and size differences between subjects were removed by scaling, translation and rotation (Procrustes transformation) of the point coordinates. The constructs of a previous model, built from sagittal MRI data (L1-S1) of 30 asymptomatic volunteers aged 20–52 (mean 29, standard deviation (SD) 9.6) years [14], were used to enable consistency in the shape variations described (shape ‘modes’). The original mode score distributions were normalized to have a mean of zero and unit standard deviation so that the unit of measurement was in standard deviations. Thus, the mode scores for individuals in the current cohort were calculated in relation to those of a healthy asymptomatic cohort.

### Statistical analysis

Statistical analysis was undertaken using SPSS software (Version 24, IBM SPSS statistics, IBM Corp.). The normality of the data was assessed using frequency histograms, quantile-quantile plots and the Shapiro-Wilk test. To assess preliminary trends in the data, associations between LDD (LDD sum scores), age, BMI and intrinsic shape (mode scores) were established using Pearson correlations. Levene’s Test confirmed homogeneity of variance. One-way repeated-measures analysis of variance (ANOVA) was used to assess the differences between the LDD groups with Games Howell (for samples with unequal variance) or Hochberg’s T2 (for samples with equal variance but unequal sample sizes) post-hoc comparisons. In the case of unequal variance Welch’s corrected F-ratio was reported. The observed power and effect sizes were also computed (*r* =  √ η^2^, where *r* = effect size and η^2^= eta squared or SS_M_ (the between group effects)/ SS_T_ (the total amount of variance in the data)). The effects of potential confounding variables such as age, BMI and sex were explored using Pearson’s r correlation coefficient (r_p_) and independent sample *t*-tests. Results were considered significant at *P* < 0.05 for all tests. Missing data were excluded case-wise from the analysis and were not replaced by imputed values.

## Results

**Fig. 2:**
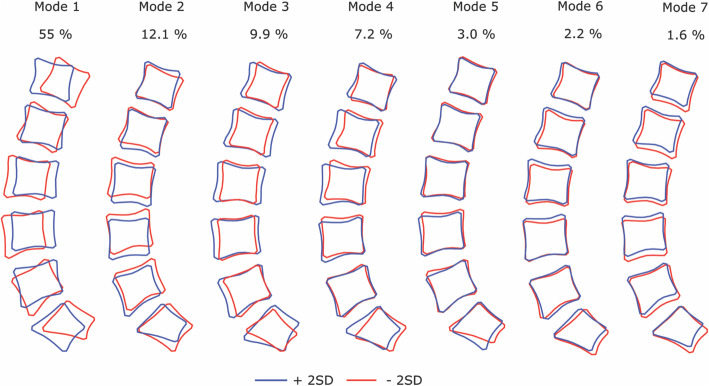
The modes of sagittal lumbar variation and percentage variance. Mode 1 represents curviness, Mode 2 evenness, Mode 3 vertebral depth, Mode 4 evenness and reduced intervertebral space (L4-S1), Mode 5 represents changes in sacrum morphology, Mode 6 describes variations in vertebral shape and intervertebral spaces (L4-S1) and Mode 7 reduced intervertebral spaces (L4-S1). Blue corresponds with + 2 standard deviations (+2SD) and red with − 2 standard deviations (− 2SD))

Seventy participants completed this study (31 male, 39 female, mean age 49 years (SD 11, range 31–71 years), BMI mean 26 kg m^− 2^ (SD 5). Ninety one percent of the variation in participant lumbar shape was explained by the first seven modes (M1 – M7) in descending order of variance (Fig. 2) [11]. Modes contributing to greater than 1.5% of the variance observed (≤M8) were chosen according to the scree plot.

**Fig. 3:**
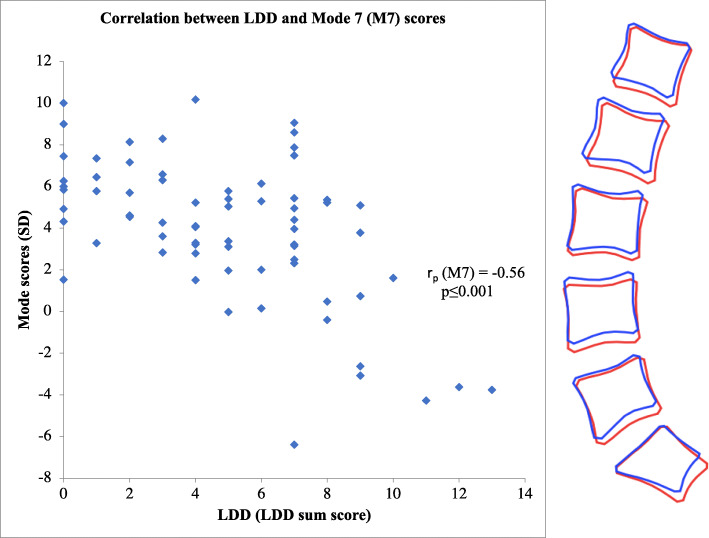
LDD correlates with intrinsic shape (Mode 7 (M7)). The figure (left) represents a negative correlation between M7 scores (SD) and LDD (r_p_ = −0.56, *p* ≤ 0.001). The figure (right) is a graphical representation of this; the greater degree of LDD the smaller the intervertebral spaces (L4-S1) (from blue (+ 2SD) to red (− 2SD)). SD-standard deviation

LDD sum scores significantly correlated with age (r_p_ = 0.51, *P* ≤ 0.0001). There was no difference in LDD sum scores between sexes (*P* = 0.60). LDD sum scores did not correlate with BMI (r_p_ = − 0.04, *P* = 0.76). Higher LDD sum scores were significantly associated with a more curvy or lordotic spine (corresponding to negative M1 scores, r_p_ = − 0.28, *P* = 0.02), a more evenly distributed lumbar curve (negative M2 scores, r_p_ = − 0.24, *P* = 0.05), larger anterior-posterior (A-P) vertebral diameter relative to vertebral height (positive M3 scores r_p_ = 0.32, *P* = 0.007) and smaller L4-S1 intervertebral disc spaces (negative M7 scores, r_p_ = − 0.56, *P* ≤ 0.001) (Fig. 3).

Four groups were identified based upon the presence or absence of LDD and recurrent LBP; ‘LDD pain’ (*n* = 24), ‘LDD no pain’ (*n* = 24), ‘No LDD no pain’ (*n* = 19) and ‘No LDD pain’ (*n* = 3) (Table 2). Although there was no significant difference in age between groups (F = 2.51, *P* = 0.07), there was a significant difference in BMI (F = 3.24, *P* = 0.03).

**Table 2:** Participant demographics

Groups	N	Age (years)(SD)	Age range (years)	BMI (kg/m^**2**^) (SD)	Gender (M/F)
**No LDD no pain**	19	45 (10)	31–69	24 (4)	6 M, 13 F
**LDD no pain**	24	52 (11)	31–74	25 (3)	11 M, 13 F
**LDD pain**	24	50 (11)	32–73	28 (5)	14 M, 7 F
**No LDD pain**	3	39 (3)	36–42	32 (1)	3 F

As directed in guidance, Table 3 is greater than one A4 page and is, therefore, located for reference at end of this document. Following production it should appear following Table 2 in this location.

**Fig. 4:**
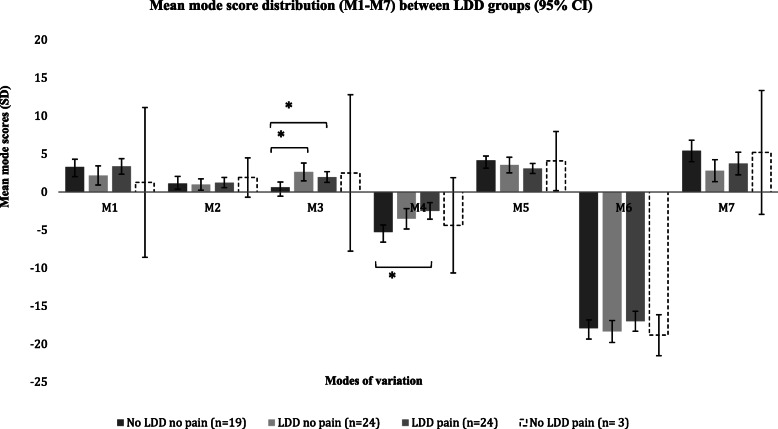
Mean intrinsic shape (mode score) distribution between LDD groups. *indicates p value ≤0.05. Bars with dashed outline (−-) indicate smallest group size, where confidence intervals are expectedly larger in the smallest group (‘no LDD and pain’ group (*n* = 3))

**Fig. 5:**
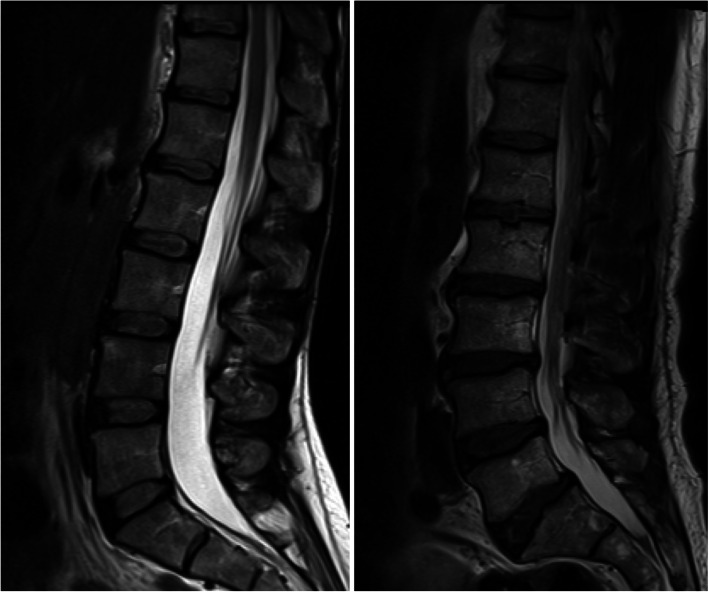
Mid sagittal T2 weighted MRI scans indicating differences in the M3 phenotype between participants. The subject on the right (‘LDD pain’ group, LDD sum score = 7) has larger a-p lumbar vertebral diameters (relative to vertebral height) than the subject on the left (‘No LDD no pain’ group, LDD sum score = zero)

Analysis of variance revealed a significant difference in the M3 phenotype (F = 3.892, *P* = 0.05 ,η^2^ =0.13, α = 0.05, observed power = 0.72) between groups. M3 corresponded to changes in A-P vertebral diameter relative to vertebral height. Post-hoc tests showed that ‘LDD no pain’ (95% CI = -2.61 to − 0.08; *P* = 0.03) and ‘LDD pain’ groups (95% CI = -3.77 to − 0.27; *P* = 0.02) had significantly larger A-P vertebral diameters relative to vertebral height than ‘no LDD no pain’ groups (Table 3, Figs. 4 & 5). This suggested an association between M3 and LDD and/or recurrent LBP. Males had significantly higher M3 scores (*P* = 0.001) indicating a larger A-P vertebral diameter. Higher BMI was also associated with larger A-P vertebral diameters (positive M3 scores, r_p_ = 0.26, *P* = 0.03) but not age (r_p_ = 0.17, *P* = 0.20). These results supported an association between the M3 differences observed between LDD groups and BMI and sex.

**Table 3:** LDD group descriptives for modes of variation (M1-M7)

Modes	Groups	N	Mean	SD	SE	95% Confidence Interval for Mean		Min	Max
						Lower Bound	Upper Bound		
**M1**	No LDD no pain	19	3.31	2.11	0.48	2.29	4.32	−2.10	5.71
	LDD no pain	24	2.18	2.99	0.61	0.92	3.45	−3.88	7.63
	LDD pain	24	3.38	2.40	0.49	2.37	4.40	0.07	8.31
	No LDD pain	3	1.27	3.97	2.29	−8.59	11.13	−2.31	5.54
**M2**	No LDD no pain	19	1.13	1.94	0.44	0.19	2.06	−4.62	4.13
	LDD no pain	24	0.99	1.77	0.36	0.24	1.74	−3.14	3.86
	LDD pain	24	1.25	1.60	0.33	0.58	1.93	−2.50	3.79
	No LDD pain	3	1.92	1.04	0.60	−0.67	4.50	1.09	3.09
**M3**	No LDD no pain	19	0.63	1.42	0.33	− 0.06	1.32	−1.59	3.51
	LDD no pain	24	2.65	2.74	0.56	1.49	3.81	−1.53	8.49
	LDD pain	24	1.97	1.67	0.34	1.27	2.68	−1.08	4.81
	No LDD pain	3	2.51	4.14	2.39	−7.78	12.80	−1.72	6.55
**M4**	No LDD no pain	19	−5.24	1.89	0.43	−6.15	−4.33	−9.34	−2.98
	LDD no pain	24	−3.51	3.18	0.65	−4.85	−2.16	− 9.52	1.23
	LDD pain	24	−2.48	2.57	0.52	−3.56	−1.39	−6.66	3.50
	No LDD pain	3	−4.37	2.52	1.45	−10.62	1.89	−6.40	−1.55
**M5**	No LDD no pain	19	4.16	1.18	0.27	3.59	4.73	1.50	6.21
	LDD no pain	24	3.57	2.42	0.49	2.55	4.59	−0.09	8.19
	LDD pain	24	3.11	1.53	0.31	2.46	3.76	0.19	5.39
	No LDD pain	3	4.09	1.57	0.91	0.19	7.98	2.28	5.07
**M6**	No LDD no pain	19	−17.89	2.22	0.51	−18.96	−16.82	−22.77	−14.17
	LDD no pain	24	−18.33	3.43	0.70	−19.78	−16.88	− 25.48	−12.81
	LDD pain	24	−17.00	3.11	0.63	−18.32	−15.69	−22.79	− 11.21
	No LDD pain	3	−18.83	1.08	0.62	−21.51	−16.14	−20.07	−18.14
**M7**	No LDD no pain	19	5.45	2.83	0.65	4.08	6.81	−3.62	10.00
	LDD no pain	24	2.82	3.43	0.70	1.37	4.26	−6.40	9.06
	LDD pain	24	3.76	3.53	0.72	2.27	5.25	−4.27	10.17
	No LDD pain	3	5.21	3.28	1.89	−2.93	13.36	3.28	9.00

Mean mode scores are presented for each LDD group with corresponding standard deviation (SD), standard error (SE) and confidence intervals. Post hoc tests indicated significant differences in mean mode 3 scores (M3) between ‘No LDD no pain’ and ‘LDD no pain’ groups and between ‘No LDD no pain’ and ‘LDD pain’ groups. Significant differences in mean mode 4 scores (M4) were also demonstrated between ‘No LDD no pain’ and ‘LDD pain’ groups

**Fig. 6:**
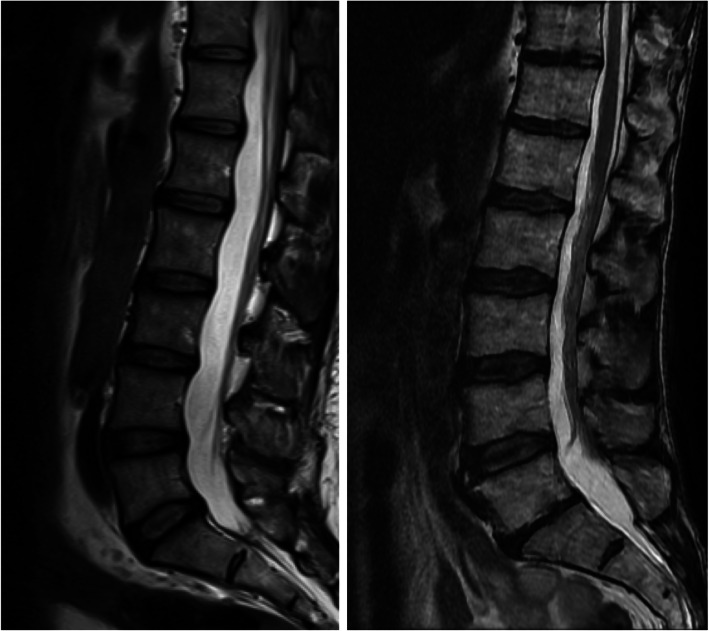
Mid sagittal T2 weighted lumbar MRI scans indicating differences in the M4 phenotype between participants. The subject on the right (‘LDD pain’ group, LDD sum score = 7) has a more evenly distributed curve with smaller L5/S1 intervertebral disc space (L5/S1 disc is darker, dehydrated and appears narrower) than the subject on the left (‘No LDD no pain’ group, LDD sum score = zero)

Analysis of variance between groups also uncovered a significant difference in M4 (F = 3.967, *P* = 0.01, η^2^ =0.15, α = 0.05, observed power = 0.81) that was not described through direct correlation with LDD alone. M4 described variation in the evenness of the lumbar curvature occurring together with changes in L5-S1 intervertebral disc space. Differences in M4 were not explained by sex *(P* = 0.20), BMI (r_p_ *= 0.20, P* = 0.20) or aging (r_p_ *= 0.30, P* = 0.05). Post-hoc comparisons revealed that the significant differences lay between ‘LDD pain’ and ‘no LDD no pain’ groups (95% CI = -4.96 to − 0.57; *P* = 0.007) (Table 3, Figs. 4, 5 and 6); the ‘LDD pain’ group had a more even lumbar curvature with smaller and less wedged L5/S1 disc spaces (positive M4). This indicated that in the presence of recurrent LBP, LDD was associated with a significant difference in the M4 phenotype.

The M4 phenotype, identified as significantly different between LDD groups, was also significantly associated with quality of life in LDD patients, specifically bodily pain, vitality, social function and mental health (SF-36) (Table 4). Intrinsic shape did not correlate with measures of self-reported function (ODI), depression or anxiety in patients with recurrent LBP.

**Table 4:** Association between M3 and M4 phenotypes and clinical outcomes

MODES		ODI	HADS		SF-36							
			D	A	PF	RF	Bodily Pain	GH	Vitality	Social Function	RE	Mental Health
**M3**	Coefficient	0.06	0.18	0.25	−0.06	−0.25	− 0.02	0.85	− 0.20	0.34	− 0.41	−0.16
	*p* value	0.78	0.41	0.27	0.78	0.26	0.93	0.70	0.37	0.88	0.06	0.46
	N	26	22	22	23	23	23	23	23	23	23	23
**M4**	Coefficient	−0.37	−0.03	−0.37	0.32	0.36	0.60**	0.39	0.43*	0.61^**^	0.38	0.55^**^
	p value	0.06	0.13	0.09	0.14	0.09	0.003	0.70	0.04	0.002	0.08	0.006
	N	26	22	22	23	23	23	23	23	23	23	23

*ODI* Oswestry Disability Index, HADS = *D* Depression, *A* Anxiety, *PF* Physical Function, *GH* General Health, *RF* Role Physical, *SF* Social Function, *RE* Role Emotional. Coefficient = Pearson’s correlation. *Significance *p* < 0.05 **Significant *p* < 0.001.

## Discussion

SSM has been used in recent studies to successfully detect healthy morphological changes associated with aging, functional activity, osteoporosis and osteoarthritis [15–18]. However, this is the first time that SSM has been used to explore associations between intrinsic spine shape variations and LDD. The results suggest that intrinsic lumbar shape is associated with LDD and quality of life in adults. Therefore, it seems that SSM could be used in future longitudinal research to explore factors associated with LDD modifiers, risk or prognosis.

In this current study, LDD was associated with a more lordotic or ‘curvy’ lumbar spine (M1), suggesting a direct correlation between LDD and lumbar lordosis. This association has been previously documented and has been attributed to a decrease in multifidus density [19]. However, it is of interest in this current study that LDD, in the absence of frank neural compression, correlated with ‘curviness’ (increased lumbar lordosis), since lumbar disc herniation (disc prolapse with neural compression) is known to be characterised by a flattened lumbar lordosis [20]. A possible explanation for this finding is that herniation with concomitant neural compression results in a protective response during which the lumbar spine flattens. The arch-model of the spine predicts that a flatter spine is stiffer due to a greater follower load and this would agree with the lower flexibility experienced by patients with advanced degenerative conditions [21]. It is unclear, however, whether the lumbar lordosis increases as LDD progresses or whether a larger lordosis is a risk factor for LDD. Further, longitudinal studies will be necessary to explore this.

LDD also correlated with a more evenly distributed lumbar curve (M2), increased A-P relative diameter of lumbar vertebrae (M3) and smaller L4-S1 intervertebral spaces (M7). Since LDD has been previously associated with disc dehydration and reduced disc height, findings of reduced distal intervertebral spaces seem representative of the condition under investigation [22, 23]. Overall, given the sparsity of high quality, longitudinal studies, it remains challenging to determine true causality.

Analysis of variance between LDD groups established an association between LDD and larger A-P relative diameters. However, M3 was also found to be significantly associated with sex (males had significantly larger vertebral depths) and BMI. This finding is unsurprising in light of the known significant association between degeneration and genetics [2] and previous SSM research which has found positive correlations between M3, BMI and sex [15]. Furthermore, since an increased risk of spondylolysis has been recently associated with smaller A-P cross sectional areas [24], it is possible that increasing A-P diameters may also represent a risk factor for LDD and reduced lumbar mobility.

However, the M4 phenotype, which corresponded to a greater evenness of the lumbar curvature and the smaller, less wedge-shaped, L5-S1 intervertebral spaces, was significantly associated with LDD in patients with recurrent LBP. As a preliminary step towards determining the potential for patient impact, this exploratory study also found new evidence to suggest an association between intrinsic shape phenotypes and self-reported clinical outcomes; the M4 phenotype also correlated significantly with features that affect patient quality of life, specifically bodily pain, social functioning and mental health. This finding is in agreement with previous studies in adults with degenerative changes which also found significant associations between posture and quality of life using the SF-36 [25, 26]. Therefore, the M4 phenotype warrants further investigation as a biomarker of LDD, particularly given the association with patient quality of life. In future such phenotypes could be used to stratify treatment for patients with degenerative conditions, thus ensuring that the right patient receives the right care.

In light of the established association between Modic change and low back pain [27], a preliminary analysis of intrinsic shape phenotypes was also undertaken using the Modic subclassification system in this cohort. Participants were assigned to each group based on the presence or absence of Modic changes and/ or pain. SSM analysis revealed that both the M3 (*p* = 0.03) and M4 (*p* = 0.03) phenotypes were similarly significant irrespective of the method of subclassification used, reinforcing the known association between LDD and Modic changes [28].

The assessment of supine MRI scans is a limitation in this current study. However, a standardised supine position was adopted so that routine NHS MRI scans could be directly compared with those of healthy controls. Previous SSM research has shown a correlation between intrinsic lumbar shape in supine, sitting and standing positions [11]. This means that an element of intrinsic spine shape, or an individual’s ‘spinal signature’, is conserved despite the position adopted [14]. It is also important to note that this signature affects the response to loading and the natural style adopted for lifting. The most lordotic spines become more lordotic under load whereas straighter spines or those with a moderate lordosis become straighter [7]. When lifting a weight from the floor, those with curvier spines prefer to flex whereas those with straighter spines prefer to squat and find flexing difficult [16]. Therefore, one can assume that any shape differences identified in this supine study would be further enhanced in standing and may affect the biomechanics of the lumbar spine.

It is acknowledged that the results of this exploratory, cross-sectional work are not generalisable and do not establish causality and as such should be interpreted judiciously. Firstly, the strategic recruitment of a specific number of participants to each group was not possible since the assignment to each group was dependent on the MRI outcome and strict inclusion criteria. Secondly, the results from the model used in this current study cannot be directly compared with models constructed from different images. It is possible that larger mode scores (SDs) reflected differences between the healthy adult model (mean 29 years) and the older adult cohort used in this study; LDD was found to be significantly correlated with age.

## Conclusions

This exploratory SSM study provides new evidence that there is an association between intrinsic lumbar shape, LDD and quality of life in patients. This highlights the potential role of SSM and intrinsic shape in the identification of risk, prognostic or potentially modifiable factors in LDD patients with recurrent pain. Further longitudinal research will be required to characterise the spine over time and establish true causality.

## Data Availability

The datasets for this current study are available from the corresponding author upon reasonable request.

## Abbreviations

ANOVA: Analysis of Variance
A: Anxiety
BMI: Body Mass Index
CI: Confidence Interval
D: Depression
GH: General Health
HADS: Hospital Anxiety and Depression Scale
ICC: Intra-class Correlation Coefficients
LBP: Low Back Pain
LDD: Lumbar Disc Degeneration
MRI: Magnetic Resonance Imaging
NHS: National Health Service
ODI: Oswestry Disability Index
PF: Physical Function
RE: Role Emotional
RF: Role Physical
SD: Standard Deviation
SF: Social Function
SF-36: Short Form-36
SSM: Statistical Shape Modelling
